# *CFH* I62V as a Putative Genetic Marker for Posner-Schlossman Syndrome

**DOI:** 10.3389/fimmu.2021.608723

**Published:** 2021-02-11

**Authors:** Ming Ming Yang, Hong Yan Sun, Ting Meng, Shan Hu Qiu, Qi Qiao Zeng, Tsz Kin Ng, Li Jiang, Ting Ming Deng, Ai Neng Zeng, Jun Wang, Xiao Ling Luo

**Affiliations:** ^1^Department of Ophthalmology, Shenzhen People's Hospital, The Second Clinical Medical College of Jinan University & The First Affiliated Hospital of Southern University of Science and Technology, Shenzhen, China; ^2^Department of Endocrinology, Shenzhen People's Hospital, The Second Clinical Medical College of Jinan University & The First Affiliated Hospital of Southern University of Science and Technology, Shenzhen, China; ^3^Joint Shantou International Eye Center of Shantou University and The Chinese University of Hong Kong, Shantou, China; ^4^Department of Ophthalmology and Visual Sciences, The Chinese University of Hong Kong, Hong Kong, China

**Keywords:** uveitis, posner-schlossman syndrome (PSS), complement system, genes, single nucleotide polymorphisms, complement factor H

## Abstract

**Objective:** Posner-Schlossman syndrome (PSS), also known as glaucomatocyclitic crisis, is an ocular condition characterized by recurrent attacks of anterior uveitis and raised intraocular pressure. Previous studies by our team and others have identified the genetic association of complement pathway genes with uveitis and glaucoma. This study aimed to investigate the complement genes in PSS patients with the view of elucidating the genetic background of the disease.

**Methods:** A total of 331 subjects (56 PSS patients and 275 controls) were recruited for this study. We selected 27 variants in six complement pathway genes (*SERPING1, C2, CFB, CFH, C3*, and *C5*) and detected them using TaqMan single nucleotide polymorphism (SNP) Genotyping Assays. Univariate SNP association analysis, haplotype-based association analysis, gene-gene interaction analysis among complement genes, and genotype-phenotype correlation analysis were performed.

**Results:** Among the 27 variants of six complement pathway genes, the functional variant I62V (rs800292) at the *CFH* gene was found to be significantly associated with PSS; there was a significant increase in the frequency of A allele and AA homozygosity in PSS patients than in controls (*P* = 1.79 × 10^−4^; odds ratio (OR) 2.18, 95% CI: 1.44–3.29; *P* = 4.65 × 10^−4^; OR 3.66, 95% CI: 1.70–7.85, respectively). The additive effect of *CFH*-rs800292 and *SERPING1*-rs3824988 was identified with an OR of 12.50 (95% CI: 2.16–72.28). Genotype-phenotype analysis indicated that the rs800292 AA genotype was associated with a higher intraocular pressure and higher frequency of recurrence. Unlike a high proportion of human leukocyte antigen (HLA)-B27 positivity in anterior uveitis, only 3 in 56 (5.36%) PSS patients were HLA-B27 positive. In addition, one haplotype block (GC) in the *SERPING1* gene showed a nominal association with PSS with an increased risk of 2.04 (*P* = 0.01; 95% CI: 1.18–3.53), but the *P*-value could not withstand the Bonferroni correction (*P*_corr_ > 0.05).

**Conclusion:** This study revealed a genetic association of a *CFH* variant with PSS as well as its clinical parameters, implying that the alternative complement pathway might play an important role in the pathogenesis of PSS. Further studies to enrich the understanding of the genetic background of PSS and the role of the complement system in ocular inflammation are warranted.

## Introduction

Posner-Schlossman syndrome (PSS), also known as glaucomatocyclitic crisis, is an ocular condition that presents with recurrent anterior uveitis and acutely with markedly elevated intraocular pressure (IOP). It is often classified as secondary inflammatory glaucoma (1, 2). The main clinical manifestations of PSS are elevated IOP and blurred vision, with a tendency to affect patients between 20 and 50 years of age. Long-term recurrent PSS patients are at a high risk of developing permanent complications, such as optic nerve atrophy and loss of vision (2).

The exact pathogenesis of PSS is largely unknown, and previous studies have suggested that genetic susceptibility, infection, and autoimmune drive may contribute to the disease (1, 3, 4). Multiple genetic loci in human leukocyte antigen (*HLA*) have been identified to be associated with PSS in Japanese and Chinese populations, such as *HLA-Ia, HLA-II, HLA-Bw54, HLA-E*, and *HLA-G*. These recent genetic studies have not only helped shed light on the pathogenesis of the disease, but also have suggested that they share common etiologies with other hypertensive uveitic conditions (5–8). To date, however, no specific gene other than *HLA* has been found to directly cause PSS.

The complement system, a key innate immune defense system, plays an important role in modulating various immune and inflammatory responses. In our previous studies, we intensively studied the genetic profiles of complement pathway genes in uveitis, including complement factor H (*CFH*), complement factor B (*CFB*), complement component 2 (*C2*), complement component 3 (*C3*), complement component 5 (*C5*), and complement component 1 inhibitor gene (*SERPING1*) (9–17). Moreover, recent studies have implicated complement cascades in glaucomatous neurodegeneration. IOP also modulates the immune system by inducing several complement components such as *CFH, C3, C1q*, and *C1r* (18, 19).

In view of the above findings and considering that PSS shared the characteristics of both anterior uveitis and glaucoma, the primary aim of this study was to investigate the genetic associations of 27 variants in complement pathway genes in PSS patients. Our secondary aim was to further identify any associations between these genes and specific phenotypes to understand the genetic background of this disease.

## Materials and Methods

### Study Participants

All study subjects were enrolled at the Chinese University of Hong Kong Eye Center and Shenzhen Peoples' Hospital. Informed consent was obtained from each study participant, and the study protocol was in accordance with the ethical guidelines of the Declaration of Helsinki. The study was approved by the Medical Ethics Committee of the Chinese University of Hong Kong Eye Center and the Clinical Research Ethics Committee of Shenzhen Peoples' Hospital.

Fifty-six PSS patients were recruited for this study. The diagnosis of PSS was based on the following clinical features: repeated episodes of unilateral moderate to high elevation of IOP with blurred vision, mild anterior chamber inflammation and mutton-fat-like keratic precipitates (KPs), open anterior chamber angles under high IOP, and no obvious posterior synechiae of the iris (20). For each patient, the clinical course of ocular inflammation was documented, including visual acuity, slit-lamp biomicroscopy, IOP, age at onset, clinical features (KPs, posterior synechiae, and anterior chamber cells), laterality, frequency of recurrence, and complications of PSS. A total of 275 individuals without any systemic immune-related disorders and major eye diseases except senile cataract were recruited as control subjects. All subjects underwent full clinical examination and basic ophthalmic investigations.

### Selection and Genotyping of Single Nucleotide Polymorphisms

Twenty-seven single nucleotide polymorphisms (SNPs) in six genes/loci in the complement pathway were selected in the present study, including rs17030, rs344555, rs2241393, rs2241392, rs428453, rs11672613, rs2230205, and rs2250656 in *C3*; rs3020644, rs9332739, rs4151667, rs1048709, rs17201431, rs537160, and rs2072633 in the *C2/CFB* locus; rs1005511 and rs3824988 in *SERPING1*; rs12237774, rs2269066, rs17611, rs1548782, rs10985126, and rs1017119 in *C5*; and rs3753394, rs800292, and rs1065489 in *CFH*. Genomic DNA was extracted from peripheral blood using the QIAamp Blood Kit (Qiagen; Hilden, Germany). The 27 SNPs were assessed using TaqMan genotyping assays (Applied Biosystems, Foster City, CA, USA) in a Roche LightCycler 480 real-time polymerase chain reaction System (Roche Diagnostics, Basel, Switzerland), according to the manufacturer's instructions. The HLA-B27 allele was detected using nested polymerase chain reaction as described by Konno et al. (21).

### Statistical Analysis

The Hardy-Weinberg equilibrium of each SNP in the controls was assessed using the χ^2^ test in PLINK (V.1.07, available in the public domain at http://pngu.mgh.harvard.edu/~purcell/plink/). Allelic and genotypic frequencies between cases and controls were compared using the χ^2^ test or Fisher's exact test. Dominant and recessive models were applied to investigate the disease association with regard to the minor allele, and the odds ratio (OR) and 95% confidence intervals (CI) were calculated. Logistic regression analysis was applied to adjust for the association of these SNPs with age and sex. IOP, age at onset, anterior chamber cells, and frequency of recurrence were analyzed by the *t*-test or Mann–Whitney *U* test, whereas the presence of posterior synechiae and KPs were analyzed by χ^2^ test or Fisher's exact test. Moreover, stratified analysis based on the clinical manifestations was also performed. Pairwise linkage disequilibrium between polymorphisms and expectation-maximization algorithm-based haplotype association analyses were performed using Haploview (ver. 4.2). Furthermore, we applied the W-test for gene-gene interaction analysis. The joint effects of these pairs of SNPs were also analyzed with reference to the individuals with both the non-risk genotypes at the two studied SNPs. In individual association analysis, a *P* < 0.05 was considered statistically significant. The Bonferroni method was used to correct the *P-*values in multiple testing, with a *P* < 0.0019 (*P* = 0.05/27, where 27 was the number of SNPs included in this study) being considered statistically significant.

## Results

### Clinical Characteristics

The demographic and clinical characteristics of the PSS patients and healthy controls are shown in Table 1. This study involved a total of 331 unrelated subjects, including 56 patients with PSS (24 males, 32 females; mean age ± SD: 49.9 ± 14.0 years) and 275 controls (122 males, 153 females; mean age ± SD: 54.3 ± 7.6 years). Among the PSS patients, 53 (94.6%) had increased IOP at first onset, 50 (89.3%) had recurrent episodes of PSS, and the average frequency of recurrence was 6.3±4.9. Forty-two (75.0%) patients had KP signs and only three (5.4%) were HLA-B27 positive.

**Table 1:** The demographic and clinical features of the PSS cases and controls.

	**Total**	**%**
**PSS patients**		
Age (year, mean ± SD)	49.9 ± 14.0	
Male	24	42.9
Female	32	57.1
Affected eyes		
Right eye	29	51.8
Left eye	25	44.6
Both eyes	2	3.6
IOP >21 mmHg	53	94.6
IOP (mmHg, mean ± SD)	42.8 ± 11.7	
KPs	42	75.0
Recurrent frequency (times, mean ± SD)	6.3 ± 4.9	
**Healthy controls**		
Age (year, mean ± SD)	54.3 ± 7.6	
Male	122	44.4
Female	153	55.6

*PSS, Posner-Schlossman syndrome; IOP, intraocular pressure, the highest IOP of the affected eye; KPs, keratic precipitates*.

### Association of Complement Genes With PSS

All the selected SNPs conformed to the Hardy-Weinberg equilibrium in the control group. Twenty-seven SNPs in *C3, C2/CFB, SERPING1, C5*, and *CFH* in the complement pathway were genotyped and statistically analyzed in PSS patients and healthy controls. Among the 27 SNPs, rs800292 in *CFB* was significantly associated with PSS (*P* = 1.79 × 10^−4^; OR 2.18, 95% CI: 1.44–3.29), with the minor allele A conferring a 2.18-fold increased risk of PSS (Table 2). Meanwhile, genetic association was also identified in the genotypic recessive model; there was a significant increase in the frequency of AA homozygosity in PSS patients than in controls (*P* = 4.65 × 10^−4^; OR 3.66, 95% CI: 1.70–7.85). In addition, a higher frequency of the *SERPING1*/rs3824988 CC genotype and C allele was found in PSS patients than in healthy controls (CC: *P* = 0.036; OR 7.73; C: *P* = 0.01, OR 1.99). A lower frequency of the *C5*/rs1017119 C allele was found in PSS patients (*P* = 0.043; OR 0.47) (Table 3). However, these two associations lost significance after adjusting for multiple testing. No significant association was identified for the other 24 SNPs, either in allelic or genotypic models. Gender stratification analysis of rs800292 showed a statistically significant association in female PSS patients (*P* = 0.001; OR 2.60, 95% CI: 1.50–4.51), but not in male patients (Table 4).

**Table 2:** Allelic association of genetic variants with PSS.

**No**.	**Gene/Locus**	**Variants**	**Minor allele**	**PSS(*n =* 56)**	**Control(*n =* 275)**	***P*-values**	**OR (95% CI)**
1	*C3*	rs17030	G	0.420	0.415	0.92	1.02 (0.07–1.54)
2		rs344555	A	0.312	0.251	0.18	1.35 (0.87–2.12)
3		rs2241393	G	0.312	0.344	0.53	1.15 (0.74–1.78)
4		rs2241392	G	0.330	0.313	0.71	1.08 (0.70–1.67)
5		rs428453	C	0.170	0.162	0.84	1.06 (0.62–1.82)
6		rs11672613	G	0.455	0.427	0.58	1.12 (0.75–1.69)
7		rs2230205	A	0.464	0.424	0.43	1.18 (0.78–1.77)
8		rs2250656	G	0.232	0.236	0.92	1.02 (0.63–1.66)
9	*C2-CFB*	rs3020644	A	0.429	0.484	0.29	1.25 (0.83–1.88)
10		rs9332739	C	0.009	0.027	0.49^*^	2.94 (0.39–22.53)
11		rs4151667	A	0.018	0.029	0.75^*^	1.65 (0.37–7.27)
12		rs1048709	A	0.317	0.280	0.44	1.20 (0.76–1.88)
13		rs17201431	C	0	0.020	0.23^*^	1.02 (1.01–1.03)
14		rs537160	A	0.491	0.484	0.89	1.03 (0.69–1.55)
15		rs4151657	C	0.277	0.251	0.57	1.14 (0.72–1.80)
16		rs2072633	G	0.393	0.435	0.42	1.19 (0.78–1.80)
17	*C1INH*	rs1005511	G	0.295	0.227	0.13	1.42 (0.90–2.23)
18		rs3824988	C	0.188	0.104	0.01	2.00 (1.15–3.45)
19	*C5*	rs12237774	T	0.152	0.198	0.25	1.38 (0.79–2.41)
20		rs2269066	C	0.170	0.218	0.25	1.37 (0.80–2.33)
21		rs17611	G	0.357	0.411	0.29	1.26 (0.82–1.92)
22		rs1548782	T	0.214	0.204	0.80	1.07 (0.65–1.75)
23		rs10985126	C	0.188	0.244	0.20	1.40 (0.84–2.33)
24		rs1017119	C	0.071	0.142	0.04	2.15 (1.01–4.58)
25	*CFH*	rs3753394	C	0.491	0.400	0.08	1.45 (0.95–2.19)
26		rs800292	A	0.491	0.307	1.79 × 10^−4^	2.18 (1.44–3.29)
27		rs1065489	T	0.482	0.442	0.15	1.35 (0.89–2.05)

*PSS, Posner-Schlossman syndrome; C3, component 3; C2, component 2; CFB, complement factor B; SERPING1, component 1 inhibitor gene; C5, component 5; CFH, complement factor H; CI, confidence interval; OR, odds ratio*;

* *Fisher exact test*.

**Table 3:** Genotypic association of rs3824988, rs1017119, and rs800292 in PSS patients and healthy controls.

**SNP**	**Genotype/Allele**	**PSS**		**Controls**		***P-*value**	**OR**	**95% CI**
		***n***	**%**	***n***	**%**			
rs3824988	CC	3	5.4	2	0.7	0.046	1.90	1.01–3.57
*SERPING1*	CT	15	26.8	53	19.3	0.036^*^	7.73	1.26–47.36
	TT	38	67.9	220	80.0			
	C	21	18.8	57	10.4	0.012	1.99	1.15–3.45
rs1017119	CC	0	0.0	5	1.8	0.052	0.46	0.21–1.02
*C5*	CT	8	14.3	68	24.7	0.594^*^	1.02	1.00–1.04
	TT	48	85.7	202	73.5			
	C	8	0.9	78	14.2	0.043	0.47	0.22–0.99
rs800292	AA	13	23.2	21	7.6	0.003	2.61	1.36–5.00
*CFH*	AG	29	51.8	127	46.2	4.65 × 10^−4^	3.66	1.70–7.85
	GG	14	25.0	127	46.2			
	A	55	49.1	169	30.7	1.79 × 10^−4^	2.18	1.44–3.29

*PSS, Posner-Schlossman syndrome; SERPING1, component 1 inhibitor gene; C5, component 5; CFH, complement factor H; CI, confidence interval; OR, odds ratio*;

* *Fisher exact test*.

**Table 4:** Polymorphisms of *CFH*-rs800292 in PSS patients and healthy controls stratified by gender.

**SNP**	**Genotype/Allele**	**PSS**		**Controls**		***P-*value**	**OR**	**95% CI**
		***n***	**%**	***n***	**%**			
Male	AA	4	16.7	10	6.5	0.085	2.47	0.86–7.04
	AG	15	62.5	64	41.8	0.248^*^	2.24	0.64–7.85
	GG	5	20.8	48	31.4			
	A	23	47.9	84	34.4	0.076	1.75	0.94–3.27
Female	AA	9	28.1	11	9.0	0.015	2.73	1.19–6.28
	AG	14	43.8	63	51.6	0.002^*^	5.05	1.89–13.53
	GG	9	28.1	79	64.8			
	A	32	50.0	85	27.8	0.001	2.60	1.50–4.51

*PSS, Posner-Schlossman syndrome; CFH, complement factor H; CI, confidence interval; OR, odds ratio*;

* *Fisher exact test*.

### Comparison of Clinical Parameters in Different Genotypes

**Figure 1:**
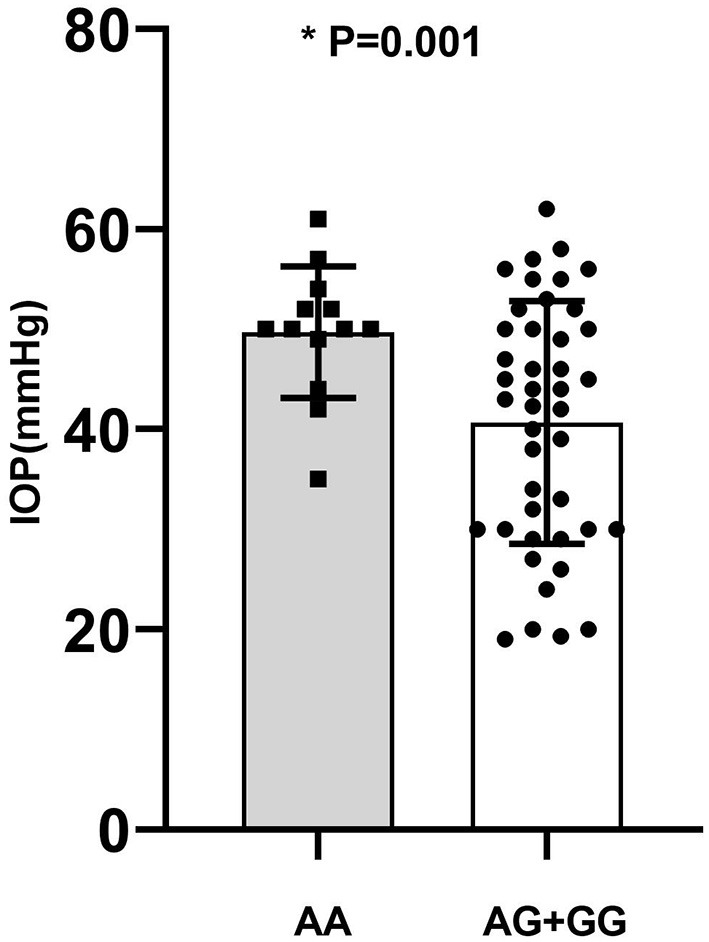
The average of IOP in PSS patients according to genotype of rs800292. AA: 49.69 ± 6.58; AG+GG: 40.67 ± 12.14 (mmHg). *statistical significance.

**Figure 2:**
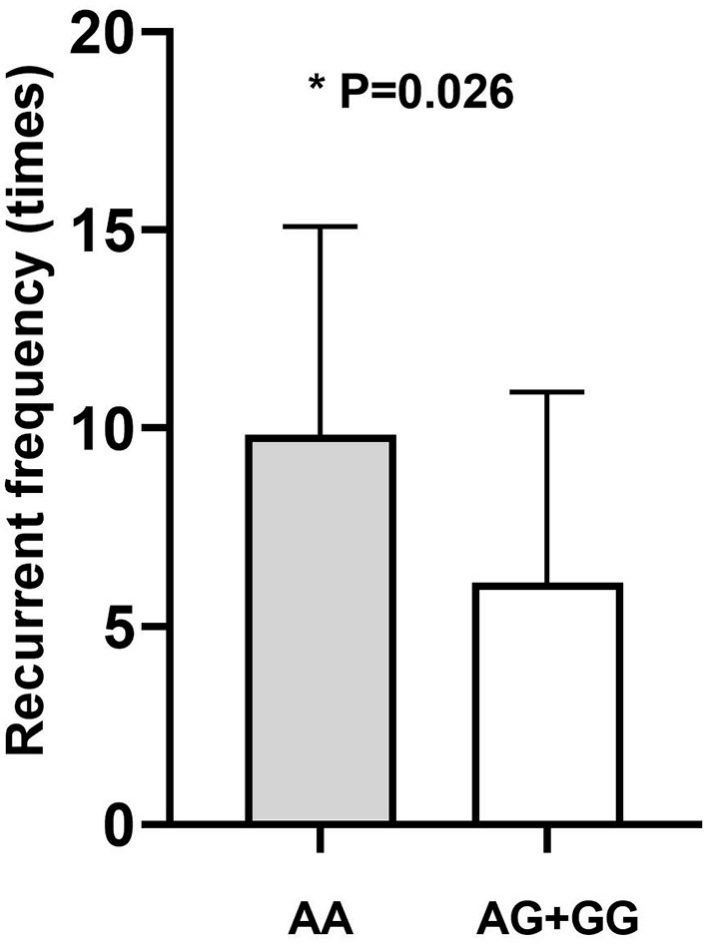
The average disease recurrent frequency of PSS according to genotype. AA: 9.83 ± 5.25; AG+GG: 6.11 ± 4.81 (times). *statistical significance.

Genotype-phenotype correlation analysis was done in terms of multiple clinical features such as IOP, age of onset, number of flares, frequency of recurrence, and the presence of KPs. Considering the significant association of *CFH*-rs800292 with PSS in this study, correlations between specific genotypes and clinical features were evaluated in the PSS patients. We discovered a relationship between rs800292 and IOP level, as well as frequency of recurrence. The mean IOP (49.69 ± 6.58 mmHg) in patients homozygous for the risk allele A at SNP rs800292 was significantly higher than patients with the genotypes AG+GG (40.67 ± 12.14 mmHg; *P* = 0.001; Figure 1). Similarly, patients with genotype AA had a relatively higher frequency of recurrence (9.83 ± 5.25 times) than those with G carriers (6.11 ± 4.81 times; *P* = 0.026; Figure 2). Associations with other clinical features were not observed.

### Gene-Gene Interaction and Joint-Effect Analysis

**Figure 3:**
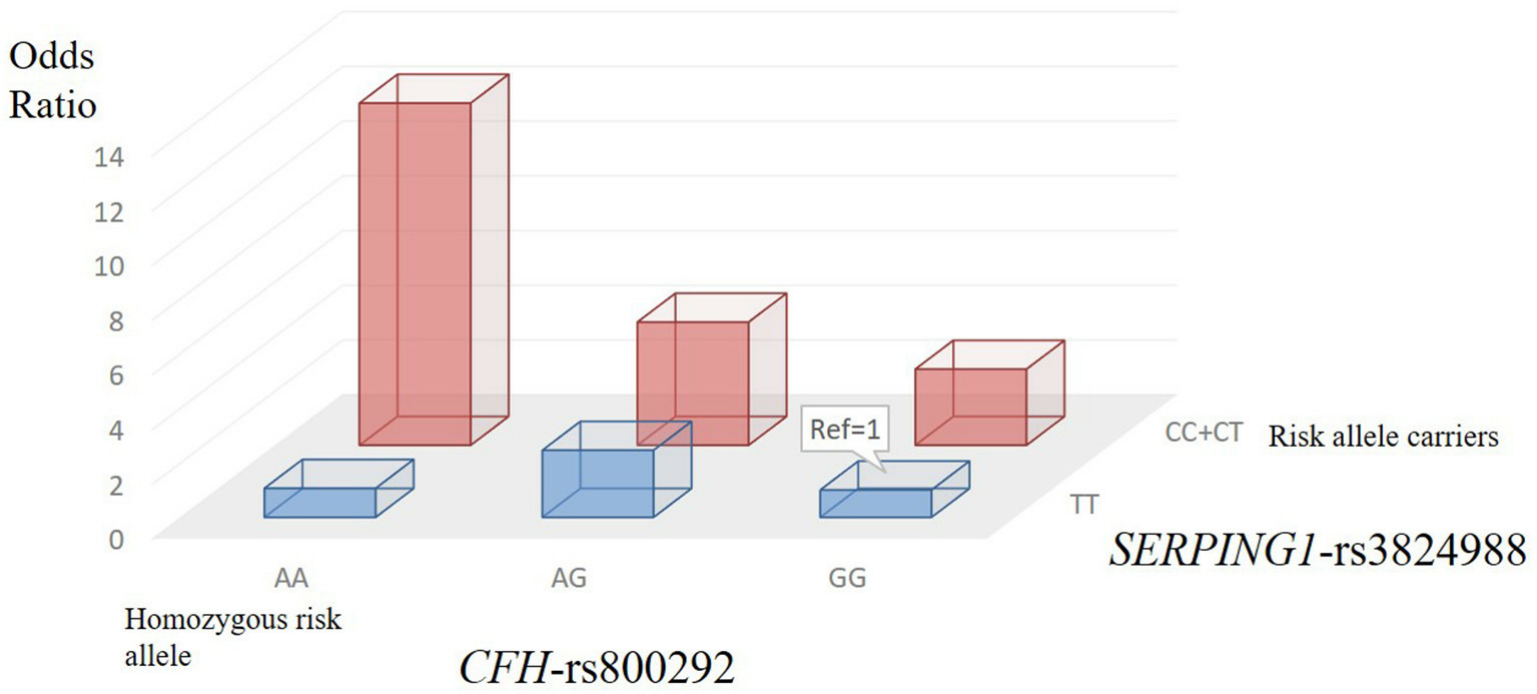
Two-locus (*CFH* and *SERPING1*) genotype-specific PSS risk.

We applied the W-test for pairwise gene-gene interaction analysis and identified a significant interaction of one pair: *CFH*-rs800292 with *SERPING1*-rs3824988. Joint-effect analysis was conducted to estimate the ORs of PSS for each possible combination of genotypes from these two loci, with the homozygous non-risk genotypes as reference. A joint disease OR of 12.50 in individuals with the rs800292 AA genotype and rs3824988 risk allele (C) carriers was observed than in the baseline non-risk genotypes, with the calculated 95% CI in a wide range from 2.16 to 72.28 (*P* = 0.012; Table 5; Figure 3)

**Table 5:** Interaction analysis between *CFH*-rs800292 and *SERPING1*-rs3824988.

**a. Genotype distribution**	***CFH*****-rs800292**					
*SERPING1*-rs3824988	**Controls (*n* = 275)**			**PSS (*n* = 56)**		
	**GG**	**AG**	**AA**	**GG**	**AG**	**AA**
TT	100 (36.3)	102 (37.1)	18 (6.5)	8 (14.3)	20 (35.7)	10 (17.9)
CT+CC	27 (9.8)	25 (9.1)	3 (1.1)	6 (10.7)	9 (16.1)	3 (5.4)
**b. Joint odds ratios and 95% confidence interval**	***CFH*****-rs800292**					
***SERPING1-*****rs3824988**	**GG**		**AG**		**AA**	
TT	1.00 (Ref)		2.45 (1.03–5.82)		1.06 (0.40–2.79)	
CT+CC	2.78 (0.89–8.69)		4.50 (1.58–12.84)		12.50 (2.16–72.28)	

*PSS, Posner-Schlossman syndrome; SERPING1, component 1 inhibitor gene; CFH, complement factor H*.

### Linkage Disequilibrium and Haplotype Association Analysis

**Figure 4:**
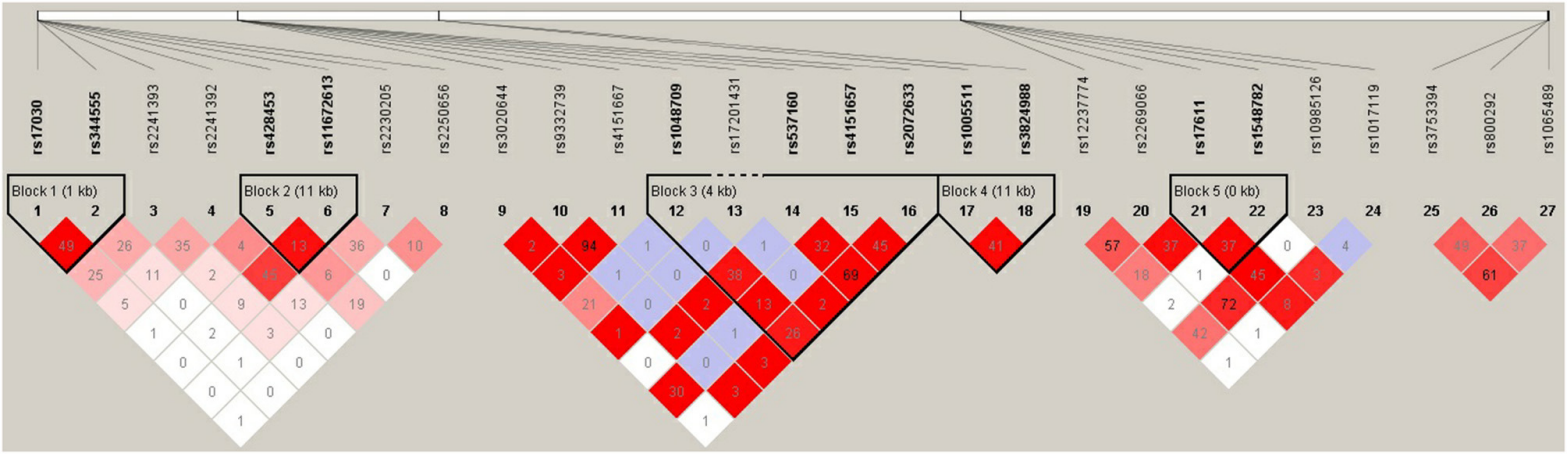
Pairwise LD among 27 SNPs in complement genes across complement system. The haplotype block was defined by the confidence interval method implemented in the Haploview software. The LD (*r*^2^) between any two SNPs is listed in the cross cells.

Pairwise linkage disequilibrium analysis was performed for all six selected genes in the complement pathway using these 27 SNPs, and five haplotype blocks were detected. Block 1 involved SNPs rs17030 and rs344555 at *C3*; Block 2 involved SNPs rs428453 and rs11672613 at *C3*; Block 3 involved SNPs rs1048709, rs537160, rs4151667, and rs2072633 at the *C2/CFB* locus; Block 4 involved SNPs rs1005511 and rs3824988 at *SERPING1*; and Block 5 involved SNPs rs17611 and rs1548782 at *C5* (Figure 4). A risk haplotype, GC, defined by block 4 was identified. It conferred a 2.04-fold increased risk of PSS, but the statistical significance did not remain after multiple corrections (*P* = 0.01; permutation *P* = 0.24; Table 6).

**Table 6:** Haplotype analysis of complement pathway genes in PSS patients and healthy controls.

		**Frequency**				
**Haplotype**	**Frequency**	**PSS**	**Controls**	***P***	***Chi square***	**Odds ratio 95% CI**
**Block 1 (rs17030, rs344555)**						
AG	0.585	0.580	0.585	0.921	0.010	-
GA	0.261	0.312	0.251	0.176	1.829	-
GG	0.154	0.107	0.164	0.131	2.278	-
**Block 2 (rs428453, rs11672613)**						
GG	0.430	0.455	0.424	0.551	0.356	-
GA	0.407	0.375	0.414	0.450	0.570	-
CA	0.161	0.169	0.159	0.785	0.074	-
**Block 3 (rs1048709, rs537160, rs4151657, rs2072633)**						
AATA	0.279	0.309	0.273	0.437	0.605	-
GGCG	0.255	0.277	0.251	0.567	0.328	-
GATA	0.204	0.181	0.208	0.520	0.415	-
GGTG	0.165	0.115	0.175	0.117	2.456	-
GGTA	0.089	0.116	0.084	0.273	1.202	-
**Block 4 (rs1005511, rs3824988)**						
AT	0.760	0.705	0.771	0.138	2.197	-
GT	0.123	0.107	0.126	0.592	0.287	-
GC	0.116	0.187	0.102	0.010	6.639	2.04 (1.18–3.53)
**Block 5 (rs17611, rs1548782**)						
AA	0.596	0.643	0.587	0.274	1.199	-
GT	0.204	0.214	0.201	0.763	0.091	-
GA	0.198	0.143	0.209	0.109	2.576	-

*PSS, Posner-Schlossman syndrome; CI, confidence interval*.

## Discussion

In this study, we identified the functional variant rs800292 (I62V) at *CFH* involved in the complement system to be significantly associated with PSS (*P* = 1.79 × 10^−4^), conferring a 2.18-fold increase in risk of PSS. Such an association was also found in the genotypic dominant and recessive models. In particular, rs800292 showed a significant association with the IOP level and frequency of recurrence (*P* = 0.001 and 0.026, respectively). Patients with the risk genotype AA had a higher IOP level and higher frequency of recurrence, indicating an association with severity of inflammation and disease prognosis. Furthermore, we identified significant interactions between *CFH* rs800292 and *SERPING1* rs3824988. These findings confirmed *CFH* as a susceptibility gene for PSS, and for the first time, revealed *CFH* rs800292 (I62V) as a putative genetic marker for PSS.

PSS, also known as glaucomatocyclitic crisis, is an ocular condition that presents with markedly elevated IOP and anterior uveitis. It generally presents with unilateral blurred vision and eye discomfort; however, there are reported cases of bilateral PSS (2). In our cases, two in 56 (3.6%) had bilateral involvement, the majority of patients presented with a recurrent course, and the average frequency of recurrence was 6.3 ± 4.9 times. Although the exact mechanism remains uncertain, it has been accepted that infection and autoimmune drive may contribute to the disease. In addition, identification of the HLA locus in PSS implies a role for genetic variants of immune regulation in PSS pathogenesis (5, 7, 8). However, no specific genes other than HLA were identified to be significantly associated with PSS.

Our previous studies have made significant advancements in depicting the genetic profiles of complement pathway genes in uveitis, and *CFH* has been identified to be significantly associated with anterior uveitis as well as intermediate and posterior uveitis entities (13, 14, 16). Furthermore, *CFH* gene polymorphisms are also associated with multiple inflammatory diseases, such as age-related macular degeneration, diabetic retinopathy, and atypical hemolytic-uremic syndrome (22–24). Here, we identified the association between rs800292 at *CFH* and the clinical parameters of PSS. The complement system is a key component of innate immunity and is involved in modulating various immune and inflammatory responses. SNP rs800292 is a functional variant located in the *CFH* gene which is a major soluble inhibitor of the complement alternative pathway. Changes in the rs800292 nucleotide results in the synthesis of isoleucine instead of valine at codon 62 (I62V). This might lead to structural changes affecting the ability of C3b binding and reduced activation of the alternative pathway C3-convertase (C3bBb) (25). This subsequently causes excessive activation of the complement system to induce immunologic disorders.

We identified genotype-phenotype correlations between *CFH* rs800292 (I62V) and PSS. The functional variant conferred a higher risk for elevated IOP (*P* = 0.001) and higher frequency of recurrence (*P* = 0.026). Clinically, patients with higher IOP and more recurrent episodes have more visual complications and are at risk of developing permanent complications such as optic nerve atrophy and loss of vision (26). Therefore, rs800292 identified in this study might be a useful genetic biomarker for the prediction of severe courses and may provide guidance for the frequency of follow-up.

Furthermore, the female-specific association and SNP-gender interaction between rs800292 and PSS suggested that additional risk factors may be required for *CFH* to exert its effect on the pathogenesis of PSS. Gender differences in inflammation susceptibility have been found in previous studies by our team and others (13, 14, 27). The di?erence in susceptibility to microbial infection between males and females may be partially explained by the di?erence in sex hormones, or likely through an epistatic function (28). However, the exact mechanism is still elusive, and further validation in a larger cohort of PSS is warranted.

Considering the biological relevance of these genes in the complement cascade, we performed pairwise gene-gene interaction analysis and identified significant interactions of one particular pair: *CFH*-rs800292 with *SERPING1*-rs3824988. CFH and *SERPING1* proteins are key regulators of the alternative and classical complement pathways respectively, the protein encoded by *SERPING1* regulates complement component 1 (*C1*) by inhibiting the proteolytic activity of its subcomponents C1r and C1s (29). *SERPING1* has been shown to be associated with age-related macular degeneration and multiple immune-mediated diseases; *SERPING1* mRNA is also expressed in both the retina and the retinal pigment epithelium-choroid layers of human donor eyes (30). In addition, a risk haplotype, GC, defined by *SERPING1* SNPs rs3824988 and rs1005511 was identified, although the statistical significance did not remain after multiple corrections. Our findings provide additional evidence for the involvement of the complement system, especially the upstream cascade, in relation to PSS. Unfortunately, the functions of these genes and proteins in PSS are yet to be elucidated.

Several limitations of our study need to be discussed. First, the relatively small sample size may lower the statistical power and may generate a type II error in our statistical analysis. To reduce the likelihood of such errors, we have increased the number of healthy controls. Second, although we analyzed 27 tag-SNPs to capture the majority of common genetic variations of the complement cascade, they may not sufficiently reflect the genetic impact of the complement system on PSS, as some identified functional SNPs conferring susceptibility to immune-related diseases were not investigated in this study because of lower minor allele frequency. Last but not least, the exact biological function of these genes/loci has not yet been reported. Therefore, further investigations of more regions in the complement cascade using a larger cohort and in other ethnic groups could help to consolidate our findings. In addition, a comprehensive evaluation of the “hot” region, especially the alternative pathway, by extensive sequencing to uncover unknown variations is worthwhile.

In summary, we have identified the association of *CFH* functional variant rs800292 (I62V) with PSS and its clinical parameters, providing new evidence to support *CFH* as a susceptibility gene for PSS. This insight would help to understand the genetic background of PSS and help in the early detection of manifestations of the disease.

## Data Availability Statement

The datasets presented in this study can be found in online repositories. The names of the repository/repositories and accession number(s) can be found in the article/Supplementary Material.

## Ethics Statement

The studies involving human participants were reviewed and approved by the Medical Ethics Committee of the Chinese University of Hong Kong and the Clinical Research Ethics Committee of Shenzhen Peoples' Hospital. The patients/participants provided their written informed consent to participate in this study.

## Author Contributions

MY, JW, and XL designed the experiments. HS, TM, AZ, TD, QZ, and LJ performed the experiments. MY, SQ, and JW performed the analysis and wrote the paper. TN and XL revised the paper. All authors contributed to the editing of the paper and to scientific discussions.

## Conflict of Interest

The authors declare that the research was conducted in the absence of any commercial or financial relationships that could be construed as a potential conflict of interest.

## Abbreviations

PSS: Posner-Schlossman syndrome
IOP: intraocular pressure
*HLA*: human leukocyte antigen
*CFH*: complement factor H
*CFB*: complement factor B
*C2*: complement component 2
*C3*: complement component 3
*C5*: complement component 5
*SERPING1*: complement component 1 inhibitor gene
KP: keratic precipitates
SNPs: single nucleotide polymorphisms
OR: odds ratio
CI: confidence interval
*C1*: complement component 1.
